# Urinary Levels of 14 Metal Elements in General Population: A Region-Based Exploratory Study in China

**DOI:** 10.3390/toxics11060488

**Published:** 2023-05-27

**Authors:** Zining Zhang, Sai Guo, Liting Hua, Beibei Wang, Qiusheng Chen, Lu Liu, Li Xiang, Hongwen Sun, Hongzhi Zhao

**Affiliations:** 1 Ministry of Education Key Laboratory of Pollution Processes and Environmental Criteria, College of Environmental Science and Engineering, Nankai University, Tianjin 300350, China; 2 School of Energy and Environmental Engineering, University of Science and Technology Beijing, Beijing 100083, China; 3 Institute of Agro-Product Safety and Nutrition, Tianjin Academy of Agricultural Sciences, Tianjin 300381, China; 4 State Key Laboratory of Environmental and Biological Analysis, Department of Chemistry, Hong Kong Baptist University, Hong Kong, China

**Keywords:** metal element, urine, human biomonitoring, influence factors

## Abstract

Metal pollution may lead to a variety of diseases; for this reason, it has become a matter of public concern worldwide. However, it is necessary to use biomonitoring approaches to assess the risks posed to human health by metals. In this study, the concentrations of 14 metal elements in 181 urine samples obtained from the general population of Gansu Province, China, were analyzed using inductively coupled plasma mass spectrometry. Eleven out of fourteen target elements had detection frequencies above 85%, namely, Cr, Ni, As, Se, Cd, Al, Fe, Cu and Rb. The concentrations of most metal elements in the urine of our subjects corresponded to the medium levels of subjects in other regional studies. Gender exerted a significant influence (*p <* 0.05) on the concentrations of Tl, Rb and Zn. The concentrations of Ni, As, Pb, Sr, Tl, Zn, Cu and Se showed significant differences among different age groups and the age-related concentration trends varied among these elements. There were significant differences in the urine concentrations of Zn and Sr between those subjects in the group who were frequently exposed to soil (exposed soil > 20 min/day) and those in the group who were not, indicating that people in regular contact with soil may be more exposed to metals. This study provides useful information for evaluating the levels of metal exposure among general populations.

## 1. Introduction

Metals, including essential and nonessential elements, are a group of substances that are widely distributed in the environment [1]. Metals typically exhibit environmental persistence, bioaccumulation and non-biodegradability. In the environment, they exist in different natural concentrations which vary considerably. They are widely used in human activities, such as industrial processes [2]. In recent years, metals have also become an environmental issue, and the strong relationships between certain metals and adverse outcomes in humans have attracted the interest of researchers [1,3]. As a manufacturing and industrial power, China is increasingly emitting metal elements into the environment in various forms, affecting human health [4,5].

A growing body of evidence confirms the toxicity of some metal species. Chronic human exposure to exceedingly small doses of these metal species is associated with adverse health effects [6,7,8]. Human exposure to As, an inorganic element, is known to affect reproductive development and induce gene mutations, which are important causes of cancer in the human body. Therefore, inorganic As is considered to be a global health risk factor [9]. Pb, one of the most common pollutants in the environment, can accumulate in bones and lead to high blood pressure, cardiovascular, kidney, hearing and dental diseases, as well as spontaneous abortion [10]. Cd may lead to cardiovascular disease, chronic kidney disease and diabetes [11]. Other studies have shown that Pb and Cd are potential neurotoxins [12,13]. Even essential elements, such as Cu, Se and Ni, which are necessary for good health [14], have also been reported to play an integral role in metal toxicity [15]. Insufficient or excessive intake of essential elements may also have harmful effects on human health [16]. The inadequate intake of metals (essential and non-essential) afflicts about 300 million people in China alone [17]. Studies have confirmed that, while exposure to certain non-essential elements can have adverse effects on human health [18,19], excessive exposure to certain essential elements such as Se and Zn can also have adverse effects [20].

Researchers in many countries have carried out studies to assess metal exposure levels in different target populations. These studies have formed the basis for further metal exposure assessments in humans and other toxicology studies [19,21,22]. In the field of environmental health, human biomonitoring is now an important tool that is used to assess the internal exposure levels of individuals—and general populations—to environmental pollutants [23,24,25]. Urine samples are usually used for clinical, environmental and toxicological studies [26]. Moreover, urine is easy to collect and is a non-invasive biomonitoring method. Some countries have performed biomonitoring of elements in urine samples, such as China [27], Germany [28], South Korea [29] and Belgium [30]. Most of these studies focused on toxic metal(loid)s, such as As, Pb and Cr. To date, there have been few studies on the levels of exposure to other non-essential and essential metals in the general population [31].

**Figure 1:**
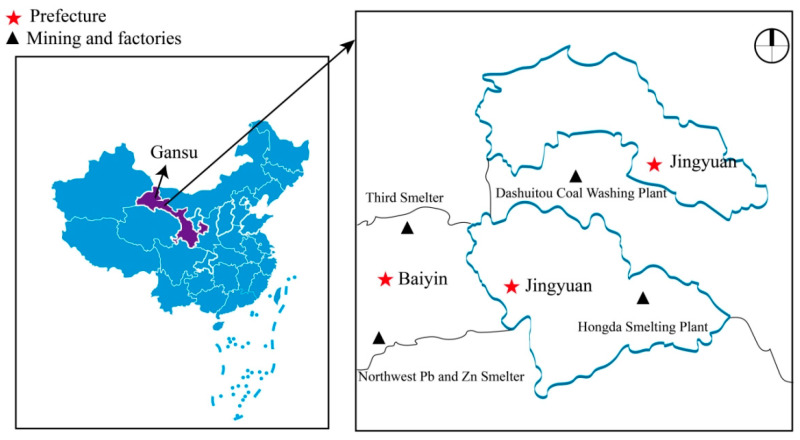
Distribution map of the sampling areas and major mining areas and factories in Gansu Province.

Gansu Province is located to the west of the Loess Plateau in Northwestern China (Figure 1). Previous studies have reported a serious metal-contamination situation in Gansu Province. Many areas in Gansu Province are rich in mineral resources, including essential and non-essential elements [32]. These areas include Jingyuan County and Baiyin City. Jingyuan County is adjacent to the downstream of Baiyin City. Baiyin City is one of the most important metal-mining and smelting bases in Northwestern China [33,34]. However, Zn and other elements in the soil of this city present a potentially high ecological risk to the local population [34]. The quality and quantity of the palygorskite deposits recently discovered in Jingyuan County mean that these reserves are now ranked among the most important globally [35]. The main chemical elements of palygorskite include Zn, Cd and Sr [36]. However, the main occupation of the population in the Jingyuan region is agricultural work. Therefore, these individuals are exposed to chemical elements in the soil which may pose a threat to the health of the local population as a whole [37]. To date, there have been no biomonitoring studies on the levels of such elements in the urine of the general population in Gansu Province; indeed, the whole issue remains unexplored. Furthermore, most current research is focused on the eastern coastal regions [19,38,39] and the most heavily metal-polluted areas [40]; in contrast, research on metal levels in the general population of Northwestern China remains limited.

In this study, therefore, we sought to measure the levels of 14 metal elements, including both non-essential elements (such as Cd, Al and Pb) and essential elements (such as Fe, Cu and Zn), in urine samples obtained from the general population in Gansu Province, China. The concentration of metal elements in urine is influenced by environmental and physiological factors. We also studied the demographic factors that might influence the levels of metal concentration, including gender, age and lifestyle. This study was based on a regional exploratory study that provided useful information for evaluating the levels of metal elements among the residents of Northwestern China.

## 2. Materials and Methods

### 2.1. Reagents and Materials

The standards for six essential elements (Ni, Se, Fe, Cu, Zn and Rb) and eight non-essential elements (Cr, As, Sr, Al, Cd, Tl, Pb and Sb) were purchased from the General Research Institute for Nonferrous Metals (Grinm, Beijing, China; 1000 μg/mL). Nitric acid (69%) was purchased from CNW (Technologies GmbH, Stuttgart, Germany). Milli-Q (18.2 MΩ·cm) ultrapure water used to prepare the samples was obtained from a Milli-Q purification system (Merck KGaA, Darmstadt, Germany).

### 2.2. Study Population and Sample Collection

The participants in the current study were all from Jingyuan County, Gansu Province, China. In total, 181 participants aged 1–74 years were recruited from eight villages in the region during 2020 (Figure 1). All subjects from Jingyuan County were Han Chinese. None of the participants were occupationally exposed to the target analytes selected in this study and none of the participants had underlying diseases. The subjects were representative of the general population in Northwestern China. The subjects used urine cups to collect 20–50 mL of middle urine, which means that the first 50 mL of urine was discarded for sampling. Middle sampling is strongly recommended in urine analysis, as contamination is the most preventable in this portion of the urine stream [41]. Participants who were required to provide urine ate a light diet the day before urine collection and then fasted overnight. Then, the specific gravity (SG) of each urine sample was measured using a digital refractometer (ATAGO, Tokyo, Japan), which was used to correct the dilution degree of urine.

Humans are exposed to elements mainly through dietary intake, respiratory tract inhalation, skin contact [1] and hand-to-mouth transmission [42]. Hand-to-mouth transmission is an important route for contact with metals, especially in infants and children [42,43]. Based on these means of exposure, the content of our study questionnaire was developed. Information on participants’ gender, height, body weight and age was obtained from this questionnaire, as well as details of their lifestyle habits (outdoor play, farm work, etc.), annual family income and other information. Informed consent was obtained from each participant, and the studies were approved by the ethics committee of Nankai University (NKUIRB2020066).

For the analysis of the obtained data, we bore in mind that the metabolism of children and adults is different, and so we divided our study subjects into four categories: young children (1–5 years, *n* = 28); children (6–11 years, *n* = 29); adolescents (12–18 years, *n* = 33); and adults (>18 years, *n* = 91). For the analysis of the relationship between soil exposure and metal concentration, we considered the different lifestyles of people of different ages, and so we also divided subjects into a lower age group (≤11 years, *n* = 58) and an older age group (>12 years, *n* = 123). We categorized age as either ≤11 years or >12 years because the latter is the cut-off age between participating and not participating in family farms in Jingyuan County. In the lower age group, participants were defined as exposed to the soil if they engaged in outdoor play for more than twenty minutes daily, or at least twice daily, in the previous year. In the older age group, participants were defined as exposed to the soil if they engaged in farm work for more than ten minutes daily, or at least once daily, in the previous year.

### 2.3. Sample Pretreatment and Instrument Analysis

All urine samples were stored at −80 °C until analysis. At the beginning of the experiment, all samples were thawed at 4 °C. Then, 1 mL of urine sample from the supernatant was added to a 15-mL CORNING centrifugal tube and acidified with 9 mL of 2% (*v*/*v*) nitric acid. Next, the resulting samples were digested by heating them at 40 °C in a water bath for one hour [44,45]. The final solution was filtered with a 0.22-μm filter (JINTENG, Tianjin, China; Polyethersulfone) for instrumental analysis.

Urinary concentrations of Cr, Ni, As, Se, Sr, Cd, Sb, Tl, Pb, Al, Fe, Cu, Zn and Rb were determined simultaneously using inductively coupled plasma mass spectrometry (ICP-MS; Agilent 7900, Santa Clara, CA, USA) [46], and analyzed in helium mode. Detailed operation conditions of ICP-MS (with collision cell) were as follows: radio frequency (RF) power, 1550 W; plasma gas flow, 15.00 L/min; auxiliary gas flow, 0.9 L/min; nebulizer flow, 1.03 L/min; atomization chamber temperature, 2 °C; sampling depth, 8 mm.

In order to check for potential contamination, procedural and solvent blanks (containing 2% nitric acid only) were prepared in each batch of 30 urine samples. A sample of 10 μL was taken from each urine sample and mixed uniformly to make a pooled QC urine sample. For each batch of 30 samples, one pooled QC urine sample was measured to check the stability of the instrument’s operation and data reliability. Germanium was used as an internal standard at a concentration of 1000 μg/L in samples and standards. The standard was added to mixed samples at low, medium or high concentrations (0.01 μg/L, 0.05 μg/L or 5 μg/L) and the analysis was repeated six times. The recovery rates of the elements were between 80.6% and 118.7%. The solvent blank was continuously determined 10 times on ICP-MS. The standard deviation value was calculated using blank measurement value analysis. The limit of detection (LOD) was calculated by multiplying the standard deviation value obtained for each element by three times [10,47]. The limit of quantitation (LOQ) was calculated by multiplying the standard deviation value obtained for each element by ten times.

### 2.4. Statistical Analysis

Urinary element concentrations were adjusted by SG using the following formula: SG-adjusted = SG-unadjusted concentration × [(SGm−1)/ (SGi−1)], where SG-adjusted is the urinary element concentration corrected by SG (μg/L), and SG-unadjusted concentration is the measured raw element concentration (μg/L). SGi is the measured SG value of the urine sample, and SGm is the median SG value in the study population.

SPSS 23 (IBM, Amenk, New York, NY, USA, 2015) software was used for statistical analysis. The element concentrations used for descriptive statistics were expressed as μg/L. Metal concentrations below the LOD were assigned with LOD divided by √2 [48,49]. The results were presented as geometric means with 95% confidence and values from the 5th to 95th percentiles. The Spearman correlation was used to analyze the correlation between any two metal elements. The Mann–Whitney U test was applied to identify differences in metal concentration and the Cu/Zn ratio between males and females, as well as between subjects who were often in contact with soil and those who were not. Cu/Zn ratio was calculated by dividing the copper (adjusted by SG) by zinc concentration (adjusted by SG) [50]. The Kruskal–Wallis test was used to compare differences in metal concentration and the Cu/Zn ratio between multiple age groups. Simultaneous pairwise comparisons and *p*-value correction were all carried out at the time of testing. All *p*-values were two-sided with a statistically significant level of 0.05.

## 3. Results and Discussion

### 3.1. Participants’ Characteristics

Demographic data for the 181 participants are shown in Table 1. The participants were 29 ± 23 years old, on average. The average body mass index (BMI) of the participants was 20.6 ± 5.3 kg/m^2^. Females represented 53.6% (*n* = 97) of the respondents. In the lower age group, 72.4% of the subjects (*n* = 42) had soil exposure as a result of playing outdoors. In the older age group, almost half of the subjects were exposed to soil due to farm work at home (49.6%, *n* = 61).

### 3.2. Urinary Concentrations of 14 Metals

The concentrations LODs and LOQs of the 14 metals in the urine of subjects are shown in Table 2 and Table S1. The geometric mean (GM) of urinary concentrations of metals (adjusted by SG) varied from 0.11 to 850.0 μg/L. With the exceptions of Sb (30.9%), Tl (68.5%) and Pb (47.0%), the detection frequencies of the remaining eleven metals were all higher than 85%. Concentrations of Sb and Pb were generally lower than their LODs. The GM concentrations of essential metals (adjusted by SG) in the urine of subjects ranged from 1.96 to 850.0 μg/L; The GM concentrations of non-essential metals ranged from 0.11 to 267.6 μg/L.

According to a document issued by the Ministry of Health of the People’s Republic of China, the concentration of Pb in urine should not exceed 120 μg/L [51]. This reference value was not exceeded in any of the samples in the current study. The US Centers for Disease Control and Prevention (CDC) [52] publishes a National Report on Human Exposure to Environmental Chemicals, which is a series of ongoing assessments of the U.S. population’s exposure to environmental chemicals carried out using biomonitoring. Samples are obtained from people who take part in CDC’s National Health and Nutrition Examination Survey (NHANES). When we compared NHANES data with the biomonitoring data of the current study, we found that urine concentrations of Cd, Cr, Pb, Ni and Sr in the 95th percentile in our study were more than twice compared to NHANES (Table S2). In addition, the urine concentration of Sr (GM concentration: 267.58 µg/L), a non-essential element, was twice as high among the Jingyuan County population compared to the NHANES subjects.

Compared with previous studies [3,39,53], the concentrations of most metals in the urine of our subjects corresponded to the medium levels of subjects in other studies (Tables S3 and S4). However, the urinary concentration levels of the non-essential element Sr (GM concentration: 267.58 µg/L; median concentration: 290.79 µg/L) and the essential element Zn (GM concentration: 397.02 µg/L; median concentration: 417.95 µg/L) in the Jingyuan County population were higher than those found in subjects from other regions in previous studies. Compared with our subjects, a population in Ethiopia [21] exhibited lower levels of Sr (GM concentration: 79.4 μg/L) exposure. Similarly, the Sr exposure level of pregnant women in Wuhan, Hubei Province, China [53] was also lower (median concentration: 239.7 µg/L). For essential elements, the Zn concentration in the urine of our study subjects was higher than that of both pregnant women in Wuhan (GM concentration: 53.3 µg/L) [54] and the general Wuhan population (median concentration: 322.9 μg/L), [53] as well as the general Ethiopian population (GM concentration: 283 μg/L) [21], indicating higher exposure in our study region. In the current study, subjects in Jingyuan County exhibited higher levels of urinary Zn and Sr. We suggest that future studies in this area should focus on these two metal elements.

**Figure 2:**
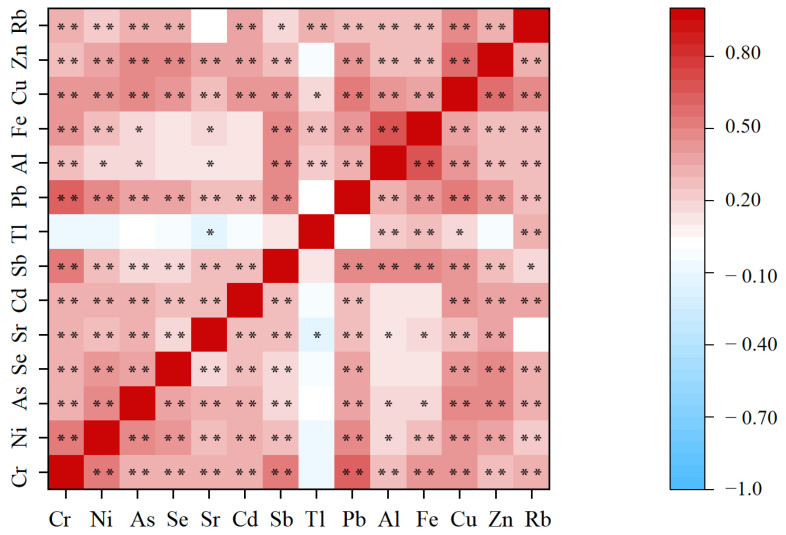
Spearman’s rank correlation coefficients between any two of 14 urinary elements. **: At *p <* 0.01, the correlation is significant. *: At *p* < 0.05, the correlation is significant.

Spearman’s rank correlation coefficients between metals were of weak-to-moderate intensity (Figure 2, r = −0.15–0.68; Table S5). Spearman’s rank correlation analyses showed significant correlations between the urinary concentrations of most metals (*p <* 0.01). Among these, there was a strong positive correlation between Fe and Al, and between Pb and Cr (reaching 0.678 and 0.634, respectively), indicating a similar pollution source. Previous studies have found that the levels of these different metals typically exhibit medium-strength correlation (r = 0.31–0.81 and −0.15–0.74, respectively) [53,55]; these results are similar to the results of the current study. Previous studies have also shown that combined exposure to multiple metals is also positively associated with increased risk of multiple human diseases [56,57]. In view of the extensive exposure of the population to various metals in the course of their daily life, further studies are needed to clarify the human health impact of such metals.

### 3.3. Factors Influencing the Element Levels in Urine

#### 3.3.1. Gender-Related Differences in Urine Element Concentrations

**Figure 3:**
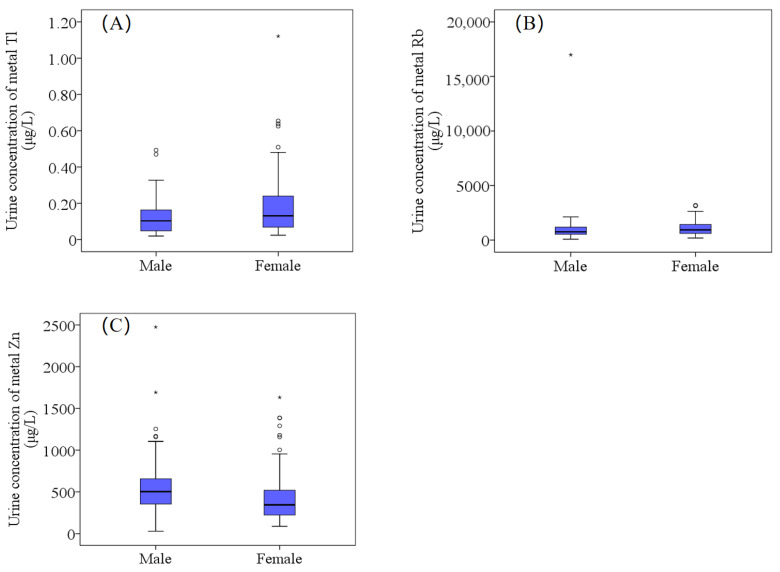
The difference in urinary concentrations of Tl (**A**), Rb (**B**) and Zn (**C**) and between males and females. There are significant differences between male and female elements in the figure; *p <* 0.05. 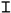: Range within 1.5. Interquartile range (IQR). ─: Median line. ○: Outlier. *: Extreme cases.

Box plots (Figure 3, Figures S1 and S2) show the differences in concentrations among different gender groups. Concentrations of Tl and Rb (Figure 3A,B, *p <* 0.05) in the urine of females (median concentrations of 0.13 μg/L and 0.93 μg/L, respectively) were significantly higher than in males (0.10 μg/L and 0.75 μg/L, respectively). Contrarily, the concentration of Zn (Figure 3C, *p <* 0.05) in the urine of males (503.1 μg/L) was significantly higher than in the urine of females (344.3 μg/L). Many studies have shown that exposure to metal elements (such as Zn, Pb and Cd) may involve remarkable gender differences [58,59,60]. Similar gender-related differences have been reported in the populations of rural areas in Ethiopia [21]. Other studies have confirmed the existence of gender differences in exposure to elements [30,61]. However, although several studies [30,61] have recorded gender dependence for biomonitoring the levels of metals, we are still uncertain about the physiological reasons for such dependence.

Furthermore, we found that the urine Cu/Zn ratio differed significantly between different genders (*p <* 0.05) (Figure S3). Overall, the urinary Cu/Zn ratios of females were slightly higher than those of males. This finding is of interest because the Cu/Zn ratio is crucial in the process of disease development [50]. Cu and Zn are present in several metal enzymes, such as the antioxidant enzyme superoxide dismutase [62]. In previous studies, a high Cu/Zn-ratio has been associated with a high risk of cardiovascular mortality [63,64], cirrhosis [62], cancer [65] and neurodegeneration [62]. It has been proposed that the Cu/Zn ratio will be a better predictor of several pathological and prepathological stages [66] because it better reflects the interaction between Cu and Zn than the concentration levels of Cu or Zn alone [67]. The roles played by the Cu/Zn ratio and by gender differences in human health need to be further investigated. In short, future studies should consider gender differences when assessing the relationship between the risk of certain diseases and Cu/Zn ratios, as well as the level of metal exposure in a study region.

#### 3.3.2. Age-Related Differences in Urine Element Concentrations

**Figure 4:**
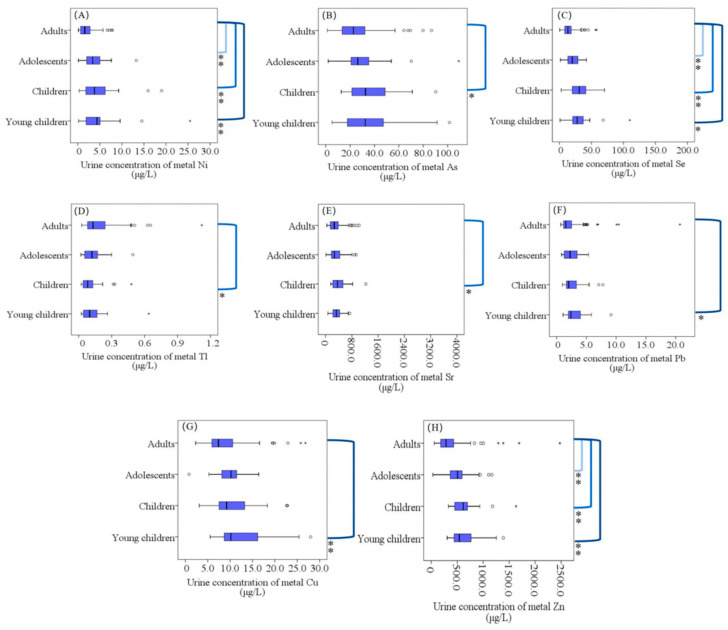
The difference in urinary concentrations (μg/L) of metals (Ni (**A**), As (**B**), Se (**C**), Tl (**D**), Sr (**E**), Pb (**F**), Cu (**G**) and Zn (**H**)) between the different age groups. Inside boxplots: 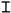: Range within 1.5. Interquartile range (IQR). ─: Median line. ○: Outlier. *: Extreme cases. Outside boxplots: **]**: Significant differences between the two groups. ✱: *p* < 0.05. ✱✱: *p* < 0.01.

Our results showed that urinary concentrations of metals, including Ni, As, Se, Tl, Sr, Pb, Cu and Zn, differed significantly across age groups (*p <* 0.05) (Figure 4 and Figure S4). In the adults group, the median concentrations of essential elements Ni, Se and Zn in the urine of participants were lower than those of the participants in the young children, children and adolescents groups (median concentrations: Table S6). It can be seen that the median concentrations of Sr and Zn in the urine of the adults group were lower than those in the children group. In addition, the median concentrations of Pb and Cu in the urine of subjects in the young children group were higher than those in the adults group.

Compared to adults and adolescents, children may have higher levels of metals in their bodies because they have a higher basal metabolic rate [68,69], a higher relative food intake and lower toxin clearance than adults. In addition, children have a much larger skin surface area per unit of body weight than adults [42,43,70]. Consequently, they can absorb more toxic substances through the skin and load more toxic substances into the body. The organs or tissues of children are still developing, so they are more sensitive to the effects of environmental pollutant metals. When interacting with the environment, children’s physical activity, including hand–mouth habits, is usually higher than that of adults [43,70]. Children are more likely to come into contact with elements through different pathways than adults, and they may receive higher doses of toxins than adults. For example, children may play with toys containing metal, resulting in exposure to higher doses of metals [71]. Children are more vulnerable than adults to exposure to elements through different routes [42] and they may receive higher doses of toxins than adults. Many recent studies have shown that heavy-metal exposure affects the health and development of children [72,73] and may impair their growth. Elements such as Pb and Cu have been reported to pose serious health risks to the local residents of Guiyu (Guangdong Province, China), especially children [43]. Guiyu is the largest electronic waste disposal site in China [4]. A previous study also claimed that Cr and Pb have been identified as the main metals responsible for the non-cancer health risks arising in children in southern Nigeria [42,74]. In this study, we proposed that different urine levels of the metal elements would affect different age groups differently; we concluded that metal elements may have especially adverse effects on the health of children.

In addition, in the current study, we found that the Cu/Zn ratio differed significantly between the adults and adolescents groups, and between the adults and children groups (*p* < 0.01) (Figure S5). Zinc is mainly excreted by pancreatic exocrine secretion, and a small fraction is excreted in the urine. Urinary zinc testing can reflect the corresponding metabolism of zinc in the body when the body is deficient in zinc [75]. It has been noted that elderly individuals are prone to mild Zn deficiency [76]. This may be the reason for the higher Cu/Zn ratio in the adults group. Due to the importance of the Cu/Zn ratio, the relationship between the urinary Cu/Zn ratio and risks to human health will be investigated in follow-up studies.

#### 3.3.3. Differences in Urine Element Concentration in Groups of People with or without Soil Contact

Every year, large quantities of metal elements are released into the environment from mining and agriculture. In Jingyuan County, the smelting and mining of non-ferrous metals in neighboring Baiyin City over the past 70 years have led to serious soil pollution with Cd, Cu, Pb and Zn [34]. Diet and skin contact are considered the major sources of exposure to elements among non-occupationally exposed human populations [1]. In addition, as mentioned above, more reserves of palygorskite have recently been found in the middle of Jingyuan County [36]. The main chemical elements that comprise palygorskite are Zn and Sr [36].

**Figure 5:**
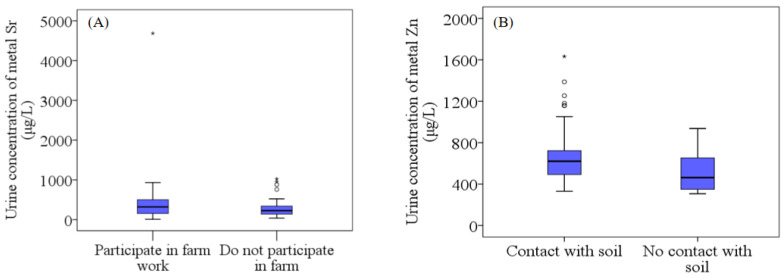
The difference in urinary concentrations of Sr (**A**) and Zn (**B**) between subjects exposed to soil and other subjects not exposed to soil. There are significant differences between the elements in the figure; *p <* 0.05. 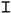: Range within 1.5. Interquartile range (IQR). ─: Median line. ○: Outlier. *: Extreme cases.

Urine concentrations of Sr in individuals engaged in farm work are shown in Figure 5A. Subjects who were over 12 years old (and, therefore, belonged to the older age group) and who often participated in farm work had a median urine concentration of 320.3 μg/L, which was significantly higher compared to other study subjects (227.3 μg/L) (*p <* 0.05). Sr is considered a potential essential element [77]. However, the long-term accumulation of potential essential elements may lead to metabolic dysfunction and harmful effects on human health [78]. Some experimental studies which sought to understand the mechanisms of the anabolic effect of Sr on bones have indicated that Sr is carcinogenic [79].

Among participants in the lower age group, there was a significant difference in urine Zn concentration between those subjects exposed to soil and those not exposed (*p <* 0.05). Due to childhood-related behaviors, such as higher hand-to-mouth contact and closer contact with substances contaminated by elements via outdoor activities [42,43], children are more exposed to the soil through skin contact and eating when playing outdoors. The urinary Zn concentration of children exposed to the soil while playing outdoors (median urine concentration: 620.3 μg/L) was higher than that of other children (median urine concentration: 463.4 μg/L), as shown in Figure 5B.

Many essential metals are naturally occurring elements, and organisms have different needs for them [77]. Higher levels of essential metals may induce acute or chronic toxicity [80]. Zn is an important nutrient for humans because it affects many biological functions, including development and reproduction [10]. However, increased exposure to Zn may result in toxicity and a number of negative consequences for health, especially in vulnerable populations such as children [81,82]. The results of this study indicate that human exposure to soil may lead to increased levels of metals in the urine. In other words, in Jingyuan County specifically, soil exposure can be considered one of the most important factors affecting the level of metal exposure in the local population. Although the concentrations of these two elements are not excessively high compared to other studies (Tables S2–S4), continuous biological monitoring should be carried out in this area to evaluate the exposure to metals and to determine the factors that affect the exposure, ensuring the health of the local population. For this reason, the impact of soil exposure on metal exposure levels among individuals in this region should be further studied in the future.

## 4. Conclusions

In this study, we measured the concentrations of 14 metal elements in urine samples obtained from the general population in Jingyuan County, Gansu Province, China. This study was limited by incomplete questionnaire information but made up for the shortcomings of biological monitoring in the region. The levels of metal elements found in the study participants were comparable to those found in other studies using subjects from different regions of the world. We found certain correlations between the urinary concentrations of most of the targeted metals. High detection frequencies indicated that such pollutant elements are widespread and related to human exposure. Metallic elements are a natural component of the lithosphere, hydrosphere and atmosphere. As a result, they are also present in living organisms, including the human body. A number of factors, including gender, age and contact with soil, exerted a significant influence on concentrations of heavy metals. This study provides useful information on the levels of elements in human urine in Jingyuan County and evaluated various influence factors for different elements in terms of differences in urine element concentrations. Thereby, it offers improved insights into the relationship between the levels of exposure to metals and influencing factors for the general population in Northwestern China and provides clues to design further large-scale metal exposure level surveys in Northwestern China.

## Figures and Tables

**Table 1:** General characteristics of the study population in Jingyuan County (*n* = 181).

Characteristics	N (%)
Gender	
Male	84 (46.4%)
Female	97 (53.6%)
Age (years)	29 ± 23
Young children (1–5)	28 (15.5%)
Children (6–11)	29 (16.0%)
Adolescents (12–18)	33 (18.2%)
Adults (>18)	91 (50.3%)
BMI (kg/m^2^)	20.6 ± 5.3
Does farm work	
Yes	61 (33.7%)
No	62 (34.2%)
Soil exposure due to playing outdoors	
Yes	42 (23.2%)
No	16 (8.8%)
Annual family income (Yuan/year)	
<25,000	47 (26.0%)
25,000–50,000	109 (60.2%)
>50,000	25 (13.8%)

**Table 2:** The distributions of urinary metal concentrations by SG correction (*n* = 181).

Urinary Elements	LOD (µg/L)	DF (%)	GM (µg/L) (95%CI)	Percentile (µg/L)				
				5th	25th	50th	75th	95th
Cr	0.63	87.8	1.13 (0.99, 1.30)	<LOD	0.72	0.99	1.59	3.55
Ni	0.25	89.0	1.96 (1.59, 2.40)	<LOD	0.93	2.54	4.47	9.30
As	0.01	100.0	24.58 (21.82, 27.70)	7.48	16.14	25.57	38.20	70.16
Se	0.76	97.8	16.4 (14.11, 18.95)	3.65	9.56	17.25	29.70	55.64
Sr	0.02	100.0	267.6 (234.7, 305.1)	70.82	164.3	290.8	432.0	807.1
Cd	0.01	98.3	0.53 (0.46, 0.62)	0.16	0.36	0.59	0.90	1.82
Sb	0.10	30.9	-	<LOD	<LOD	<LOD	0.22	3.52
Tl	0.04	68.5	0.11 (0.09, 0.12)	<LOD	<LOD	0.11	0.19	0.48
Pb	1.77	47.0	-	<LOD	<LOD	<LOD	3.03	5.81
Al	2.39	100.0	34.69 (31.03, 38.77)	12.20	21.88	33.40	50.73	154.4
Fe	1.44	100.0	38.36 (30 43, 39.53)	5.19	25.18	35.80	62.23	160.4
Cu	0.16	100.0	9.02 (8.28, 9.83)	3.77	6.68	9.03	11.88	21.10
Zn	0.40	100.0	397.0 (353.4, 446.0)	124.1	257.5	418.0	634.7	1166
Rb	0.05	100.0	850.0 (763.4, 946.4)	363.6	552.6	809.7	1310	2142

<LOD: Concentration below the limit of detection.

## Data Availability

The data presented in this study are available upon request from the corresponding author. The data are not publicly available due to the very large sizes of the chromatographic files.

## Supplementary Materials

The following supporting information can be downloaded at: https://www.mdpi.com/article/10.3390/toxics11060488/s1, Figure S1. The difference in urinary concentrations (μg/L) of essential metals (Cu: A, Fe: B, Se: C and Ni: D) between male and female; Figure S2. The difference in urinary concentrations (μg/L) of non-essential metals (As: A, Sr: B, Sb: C, Cd: D, Pb: E, Cr: F and Al: G) between male and female; Figure S3. The difference in concentration ratio of Cu and Zn in urine between male and female; Figure S4. The difference in concentration of Cr (A), Cd (B), Sb (C), Fe (D), Al (E) and Rb (F) in urine between different age groups; Figure S5. The difference in concentration ratio of Cu and Zn in urine between different age groups; Table S1. Geometric means of urinary metal concentrations in different gender and age groups of participants; Table S2. Levels of urinary elemental concentrations published by US Centers for Disease Control and Prevention; Table S3. The distributions of urinary essential elements concentrations in other studies; Table S4. The distributions of urinary non-essential elements concentrations in other studies; Table S5. Correlation (Spearman) matrix for levels of 14 tested metals in Jingyuan County subjects; Table S6. Different age groups urinary median concentrations (µg/L) of metals including Ni, As, Se, Tl, Sr, Pb, Cu and Zn [3,21,39,45,46,49,53,54,83,84,85,86].

## References

[1] Wang X., Wang B., Zhou M., Xiao L., Xu T., Yang S., Nie X., Xie L., Yu L., Mu G. (2021). Systemic inflammation mediates the association of heavy metal exposures with liver injury: A study in general Chinese urban adults. J. Hazard. Mater..

[2] Meeker J.D., Rossano M.G., Protas B., Padmanahban V., Diamond M.P., Puscheck E., Daly D., Paneth N., Wirth J.J. (2010). Environmental exposure to metals and male reproductive hormones: Circulating testosterone is inversely associated with blood molybdenum. Fertil. Steril..

[3] Zhong Z., Li Q., Guo C., Zhong Y., Zhou J., Li X., Wang D., Yu Y. (2022). Urinary heavy metals in residents from a typical city in South China: Human exposure and health risks. Environ. Sci. Pollut. Res..

[4] Song Q., Li J. (2014). Environmental effects of heavy metals derived from the e-waste recycling activities in China: A systematic review. Waste Manag..

[5] Li Y., Fang F., Lin Y., Wang Y., Kuang Y., Wu M. (2020). Heavy metal contamination and health risks of indoor dust around Xinqiao Mining Area, Tongling, China. Hum. Ecol. Risk Assess..

[6] Kim N.H., Hyun Y.Y., Lee K.-B., Chang Y., Ryu S., Oh K.-H., Ahn C. (2015). Environmental Heavy Metal Exposure and Chronic Kidney Disease in the General Population. J. Korean Med. Sci..

[7] Rokadia H.K., Agarwal S. (2013). Serum Heavy Metals and Obstructive Lung Disease Results from the National Health and Nutrition Examination Survey. Chest.

[8] Zimeri A.M., Robb S.W., Hassan S.M., Hire R.R., Davis M.B. (2015). Assessing Heavy Metal and PCB Exposure from Tap Water by Measuring Levels in Plasma from Sporadic Breast Cancer Patients, a Pilot Study. Int. J. Environ. Res. Public Health.

[9] Abdul K.S.M., Jayasinghe J.S., Chandana E.P.S., Jayasumana C., De Silva P.M.C. (2015). Arsenic and human health effects: A review. Environ. Toxicol. Pharmacol..

[10] Nasab H., Rajabi S., Eghbalian M., Malakootian M., Hashemi M., Mahmoudi-Moghaddam H. (2022). Association of As, Pb, Cr, and Zn urinary heavy metals levels with predictive indicators of cardiovascular disease and obesity in children and adolescents. Chemosphere.

[11] White A.J., O’Brien K.M., Jackson B.P., Karagas M.R. (2018). Urine and toenail cadmium levels in pregnant women: A reliability study. Environ. Int..

[12] Liu Q.L., Zhang R.Q., Wang X., Shen X.L., Wang P.L., Sun N., Li X.W., Li X.H., Hai C.X. (2019). Effects of sub-chronic, low-dose cadmium exposure on kidney damage and potential mechanisms. Ann. Transl. Med..

[13] Yuan G.P., Dai S.J., Yin Z.Q., Lu H.K., Jia R.Y., Xu J., Song X., Li L., Shu Y., Zhao X.H. (2014). Toxicological assessment of combined lead and cadmium: Acute and sub-chronic toxicity study in rats. Food Chem. Toxicol..

[14] Xu F., Song J., Li Y., Lai Y., Lin J., Pan J., Chi H., Wang Y., Li Z., Zhang G. (2021). Bioaccessibility and bioavailability adjusted dietary exposure of cadmium for local residents from a high-level environmental cadmium region. J. Hazard. Mater..

[15] Bocca B., Ruggieri F., Pino A., Rovira J., Calamandrei G., Martinez M.A., Domingo J.L., Alimonti A., Schuhmacher M. (2019). Human biomonitoring to evaluate exposure to toxic and essential trace elements during pregnancy. Part A. concentrations in maternal blood, urine and cord blood. Environ. Res..

[16] Ma J., Zhou Y., Wang D., Guo Y., Wang B., Xu Y., Chen W. (2020). Associations between essential metals exposure and metabolic syndrome (MetS): Exploring the mediating role of systemic in flammation in a general Chinese population. Environ. Int..

[17] Gailer J. (2012). Probing the bioinorganic chemistry of toxic metals in the mammalian bloodstream to advance human health. J. Inorg. Biochem..

[18] Choudhury T.R., Zaman S.Z., Chowdhury T.I., Begum B.A., Islam M.A., Rahman M.M. (2021). Status of metals in serum and urine samples of chronic kidney disease patients in a rural area of Bangladesh: An observational study. Heliyon.

[19] Zhao M., Ge X., Xu J., Li A., Mei Y., Yin G., Wu J., Liu X., Wei L., Xu Q. (2022). Association between urine metals and liver function biomarkers in Northeast China: A cross-sectional study. Ecotoxicol. Environ. Saf..

[20] Miao Y., Liu L., Liu C., Deng Y., Chen P., Luo Q., Cui F., Zhang M., Lu W., Zeng Q. (2021). Urinary biomarker of strontium exposure is positively associated with semen quality among men from an infertility clinic. Ecotoxicol. Environ. Saf..

[21] Godebo T.R., Paul C.J., Jeuland M.A., Tekle-Haimanot R. (2019). Biomonitoring of metals and trace elements in urine of central Ethiopian populations. Int. J. Hyg. Environ. Health.

[22] Vormittag E., Saldiva P., Anastacio A., Barbosa F. (2021). High levels of metals/metalloids in blood and urine of residents living in the area affected by the dam failing in Barra Longa, District, Brazil: A preliminary human biomonitoring study. Environ. Toxicol. Pharmacol..

[23] Sabbioni G., Castaño A., Esteban López M., Göen T., Mol H., Riou M., Tagne-Fotso R. (2022). Literature review and evaluation of biomarkers, matrices and analytical methods for chemicals selected in the research program Human Biomonitoring for the European Union (HBM4EU). Environ. Int..

[24] LaKind J.S., Pollock T., Naiman D.Q., Kim S., Nagasawa A., Clarke J. (2019). Factors affecting interpretation of national biomonitoring data from multiple countries: BPA as a case study. Environ. Res..

[25] Lv Y., Wei Y., Zhou J., Xue K., Guo Y., Liu Y., Ju A., Wu B., Zhao F., Chen C. (2021). Human biomonitoring of toxic and essential metals in younger elderly, octogenarians, nonagenarians and centenarians: Analysis of the Healthy Ageing and Biomarkers Cohort Study (HABCS) in China. Environ. Int..

[26] Zhang X., Cui X., Lin C., Ma J., Liu X., Zhu Y. (2017). Reference levels and relationships of nine elements in first-spot morning urine and 24-h urine from 210 Chinese children. Int. J. Hyg. Environ. Health.

[27] Cao Z., Lin S., Zhao F., Lv Y., Qu Y., Hu X., Yu S., Song S., Lu Y., Yan H. (2021). Cohort profile: China National Human Biomonitoring (CNHBM)—A nationally representative, prospective cohort in Chinese population. Environ. Int..

[28] Vogel N., Murawski A., Schmied-Tobies M.I.H., Rucic E., Doyle U., Kämpfe A., Höra C., Hildebrand J., Schäfer M., Drexler H. (2021). Lead, cadmium, mercury, and chromium in urine and blood of children and adolescents in Germany—Human biomonitoring results of the German Environmental Survey 2014–2017 (GerES V). Int. J. Hyg. Environ. Health.

[29] Lee J.W., Lee C.K., Moon C.S., Choi I.J., Lee K.J., Yi S.-M., Jang B.-K., Yoon B.j., Kim D.S., Peak D. (2012). Korea National Survey for Environmental Pollutants in the Human Body 2008: Heavy metals in the blood or urine of the Korean population. Int. J. Hyg. Environ. Health.

[30] Hoet P., Jacquerye C., Deumer G., Lison D., Haufroid V. (2013). Reference values and upper reference limits for 26 trace elements in the urine of adults living in Belgium. Clin. Chem. Lab. Med..

[31] Domingo-Relloso A., Grau-Perez M., Briongos-Figuero L., Gomez-Ariza J.L., Garcia-Barrera T., Duenas-Laita A., Bobb J.F., Chaves F.J., Kioumourtzoglou M.A., Navas-Acien A. (2019). The association of urine metals and metal mixtures with cardiovascular incidence in an adult population from Spain: The Hortega Follow-Up Study. Int. J. Epidemiol..

[32] Li Y., Gou X., Wang G., Zhang Q., Su Q., Xiao G. (2008). Heavy metal contamination and source in and agricultural soil in central Gansu Province, China. J. Environ. Sci..

[33] Nan Z., Zhao C. (2000). Heavy metal concentrations in gray calcareous soils of Baiyin region, Gansu Province, PR China. Water Air Soil Pollut..

[34] Chu Z., Lin C., Yang K., Cheng H., Gu X., Wang B., Wu l., Ma J. (2022). Lability, bioaccessibility, and ecological and health risks of anthropogenic toxic heavy metals in the arid calcareous soil around a nonferrous metal smelting area. Chemosphere.

[35] Liu L., Zhang X., Zhong T. (2016). Pollution and health risk assessment of heavy metals in urban soil in China. Hum. Ecol. Risk Assess..

[36] Shi G.F., Zhi-Guo Q.I., Han S.Y. (2007). Study on Purification of Gansu Attapulgite Clay Minerals. Bull. Chin. Ceram. Soc..

[37] Li F., Yang H., Ayyamperumal R., Liu Y. (2022). Pollution, sources, and human health risk assessment of heavy metals in urban areas around industrialization and urbanization-Northwest China. Chemosphere.

[38] Dou Y., Yin Y., Li Z., Du J., Jiang Y., Jiang T., Guo W., Qin R., Li M., Lv H. (2022). Maternal exposure to metal mixtures during early pregnancy and fetal growth in the Jiangsu Birth Cohort, China. Environ. Res..

[39] Yang D., Liu Y., Liu S., Li C., Zhao Y., Li L., Lu S. (2020). Exposure to heavy metals and its association with DNA oxidative damage in municipal waste incinerator workers in Shenzhen, China. Chemosphere.

[40] Huang M., Chen J., Yan G., Yang Y., Luo D., Chen X., He M., Yuan H., Huang Z., Lu Y. (2021). Plasma titanium level is positively associated with metabolic syndrome: A survey in China’s heavy metal polluted regions. Ecotoxicol. Environ. Saf..

[41] Morita Y., Kurano M., Tanaka M., Hisasue T., Sato M., Ono Y., Sato T., Shukuya K., Yatomi Y. (2020). Midstream urine sampling is necessary for accurate measurement of the urinary level of neutrophil gelatinase-associated lipocalin in healthy female subjects. Clin. Biochem..

[42] Ngo H.T.T., Watchalayann P., Nguyen D.B., Doan H.N., Liang L. (2021). Environmental health risk assessment of heavy metal exposure among children living in an informal e-waste processing village in Viet Nam. Sci. Total Environ..

[43] Zeng X., Xu X., Boezen H.M., Huo X. (2016). Children with health impairments by heavy metals in an e-waste recycling area. Chemosphere.

[44] Xia W., Du X., Zheng T., Zhang B., Li Y., Bassig B., Zhou A., Wang Y., Xiong C., Li Z. (2016). A Case-Control Study of Prenatal Thallium Exposure and Low Birth Weight in China. Environ. Health Perspect..

[45] Schmied A., Murawski A., Kolossa-Gehring M., Kujath P. (2021). Determination of trace elements in urine by inductively coupled plasma-tandem mass spectrometry—Biomonitoring of adults in the German capital region. Chemosphere.

[46] Xue Q., Zhou Y., Gu H.T., Xie X.Y., Hou F., Liu Q., Wu H., Zhu K.H., Wan Z.H., Song R.R. (2020). Urine metals concentrations and dyslexia among children in China. Environ. Int..

[47] Lu S., Ren L., Liu Y., Ma H., Liu S., Zhu Z., Tang Z., Kang L., Liao S. (2019). Urinary parabens in children from South China: Implications for human exposure and health risks. Environ. Pollut..

[48] Akerstrom M., Barregard L., Lundh T., Sallsten G. (2013). The relationship between cadmium in kidney and cadmium in urine and blood in an environmentally exposed population. Toxicol. Appl. Pharmacol..

[49] Barregard L., Ellingsen D.G., Berlinger B., Weinbruch S., Sallsten G. (2021). Normal variability of 22 elements in 24-hour urine samples—Results from a biobank from healthy non-smoking adults. Int. J. Hyg. Environ. Health.

[50] Wang J., Lu Y., Wang X., Zhu Q. (2010). Urinary copper/zinc ratio: A promising parameter for replacement of 24-hour urinary copper excretion for diagnosis of Wilson’s disease in children. World J. Pediatr..

[51] National Health Commission of the Peoples Republic of China. http://www.gov.cn/gzdt/2010-12/13/content_1764682.htm.

[52] Centers for Disease Control and Prevention (The U.S.) National Report on Human Exposure to Environmental Chemicals. https://www.cdc.gov/exposurereport/.

[53] Fang X., Qu J., Huan S., Sun X., Li J., Liu Q., Jin S., Xia W., Xu S., Wu Y. (2021). Associations of urine metals and metal mixtures during pregnancy with cord serum vitamin D Levels: A prospective cohort study with repeated measurements of maternal urinary metal concentrations. Environ. Int..

[54] Zhang M., Liu C., Li W., Xu X., Cui F., Chen P., Deng Y., Miao Y., Luo Q., Zeng J. (2022). Individual and mixtures of metal exposures in associations with biomarkers of oxidative stress and global DNA methylation among pregnant women. Chemosphere.

[55] Bao S., Xia W., Xu S., Li Y., Lu B., Wu S., Liao J., Liu H., Sun X., Zhou A. (2020). Multiple metal exposure and platelet counts during pregnancy: A repeated measure study. Environ. Int..

[56] Liu Y., Yu L., Zhu M., Lin W., Liu Y., Li M., Zhang Y., Ji H., Wang J. (2022). Associations of exposure to multiple metals with blood pressure and hypertension: A cross-sectional study in Chinese preschool children. Chemosphere.

[57] Zhong Q., Wu H.B., Niu Q.S., Jia P.P., Qin Q.R., Wang X.D., He J.L., Yang W.J., Huang F. (2021). Exposure to multiple metals and the risk of hypertension in adults: A prospective cohort study in a local area on the Yangtze River, China. Environ. Int..

[58] Lam P.K., Kritz-Silverstein D., Barrett-Connor E., Milne D., Nielsen F., Gamst A., Morton D., Wingard D. (2008). Plasma trace elements and cognitive function in older men and women: The rancho bernardo study. J. Nutr. Health Aging.

[59] Lockitch G., Halstead A.C., Wadsworth L., Quigley G., Reston L., Jacobson B. (1988). Age- and sex-specific pediatric reference intervals and correlations for zinc, copper, selenium, iron, vitamins A and E, and related proteins. Clin. Chem..

[60] Gade M., Comfort N., Re D.B. (2021). Sex-specific neurotoxic effects of heavy metal pollutants: Epidemiological, experimental evidence and candidate mechanisms. Environ. Res..

[61] Vahter M., Akesson A., Liden C., Ceccatelli S., Berglund M. (2007). Gender differences in the disposition and toxicity of metals. Environ. Res..

[62] Mravunac M., Szymlek-Gay E.A., Daly R.M., Roberts B.R., Formica M., Gianoudis J., O’Connell S.L., Nowson C.A., Cardoso B.R. (2019). Greater Circulating Copper Concentrations and Copper/Zinc Ratios are Associated with Lower Psychological Distress, But Not Cognitive Performance, in a Sample of Australian Older Adults. Nutrients.

[63] Leone N., Courbon D., Ducimetiere P., Zureik M. (2006). Zinc, copper, and magnesium and risks for all-cause, cancer, and cardiovascular mortality. Epidemiology.

[64] Reunanen A., Knekt P., Marniemi J., Maki J., Maatela J., Aromaa A. (1996). Serum calcium, magnesium, copper and zinc and risk of cardiovascular death. Eur. J. Clin. Nutr..

[65] Mazdak H., Yazdekhasti F., Movahedian A., Mirkheshti N., Shafieian M. (2010). The comparative study of serum iron, copper, and zinc levels between bladder cancer patients and a control group. Int. Urol. Nephrol..

[66] Laine J.T., Tuomainen T.P., Salonen J.T., Virtanen J.K. (2020). Serum copper-to-zinc-ratio and risk of incident infection in men: The Kuopio Ischaemic Heart Disease Risk Factor Study. Eur. J. Epidemiol..

[67] Martinez-Peinado M., Robles A.R., Nogueras-Lopez F., Mir M.V., Lopez M.J.O., Navarro-Alarcon M. (2018). Serum zinc and copper concentrations and ratios in cirrhotic patients: Correlation with severity index. Nutr. Hosp..

[68] Vaughan L., Zurlo F., Ravussin E. (1991). Aging and energy expenditure. Am. J. Clin. Nutr..

[69] Piers L.S., Soares M.J., McCormack L.M., O’Dea K. (1998). Is there evidence for an age-related reduction in metabolic rate?. J. Appl. Physiol..

[70] Kolo M.T., Khandaker M.U., Amin Y.M., Abdullah W.H.B., Bradley D.A., Alzimami K.S. (2018). Assessment of health risk due to the exposure of heavy metals in soil around mega coal-fired cement factory in Nigeria. Results Phys..

[71] Kamara I., Adie G.U., Giwa A.S. (2023). Total and bio-accessible toxic metals in low-cost children toys sold in major markets in Ibadan, South West Nigeria. Sci. Afr..

[72] Malin Igra A., Warnqvist A., Rahman S.M., Ekström E.-C., Rahman A., Vahter M., Kippler M. (2021). Environmental metal exposure and growth to 10 years of age in a longitudinal mother–child cohort in rural Bangladesh. Environ. Int..

[73] Zheng K., Zeng Z., Tian Q., Huang J., Zhong Q., Huo X. (2023). Epidemiological evidence for the effect of environmental heavy metal exposure on the immune system in children. Sci. Total Environ..

[74] Iwegbue C.M.A., Obi G., Emoyan O.O., Odali E.W., Egobueze F.E., Tesi G.O., Nwajei G.E., Martincigh B.S. (2018). Characterization of metals in indoor dusts from electronic workshops, cybercafes and offices in southern Nigeria: Implications for on-site human exposure. Ecotoxicol. Environ. Saf..

[75] Hongcan L. (2014). Clinical application of rapid semi quantitative detection of urine zinc and quantitative detection of blood zinc. Stud. Trace Elem. Health.

[76] Mocchegiani E., Romeo J., Malavolta M., Costarelli L., Giacconi R., Diaz L.E., Marcos A. (2013). Zinc: Dietary intake and impact of supplementation on immune function in elderly. Age.

[77] Zoroddu M.A., Aaseth J., Crisponi G., Medici S., Peana M., Nurchi V.M. (2019). The essential metals for humans: A brief overview. J. Inorg. Biochem..

[78] Barneo-Caragol C., Martinez-Morillo E., Rodriguez-Gonzalez S., Lequerica-Fernandez P., Vega-Naredo I., Menendez F.V.A. (2018). Strontium and oxidative stress in normal pregnancy. J. Trace Elem. Med. Biol..

[79] Chen L., Tang L., He J., Su Y., Cen Y., Yu D., Wu B., Lin Y., Chen W., Song E. (2012). Urinary strontium and the risk of breast cancer: A case-control study in Guangzhou, China. Environ. Res..

[80] Rosso J.J., Avigliano E., Fernández Cirelli A. (2022). Essential and non-essential metals in three lowland rivers of temperate South America (Argentina): Distribution and accumulation. J. Trace Elem. Med. Biol..

[81] dos Santos M., Soares M.C.F., Baisch P.R.M., Baisch A.L.M., da Silva F.M.R. (2018). Biomonitoring of trace elements in urine samples of children from a coal-mining region. Chemosphere.

[82] Yu Y., Zhu X., Li L., Lin B., Xiang M., Zhang X., Chen X., Yu Z., Wang Z., Wan Y. (2019). Health implication of heavy metals exposure via multiple pathways for residents living near a former e-waste recycling area in China: A comparative study. Ecotoxicol. Environ. Saf..

[83] Lee N., Wang H., Du C., Yuan T.-H., Chen C., Yu C., Chan C. (2022). Air-polluted environmental heavy metal exposure increase lung cancer incidence and mortality: A population-based longitudinal cohort study. Sci. Total Environ..

[84] Mo X., Cai J., Lin Y., Liu Q., Xu M., Zhang J., Liu S., Wei C., Wei Y., Huang S. (2021). Correlation between urinary contents of some metals and fasting plasma glucose levels: A cross-sectional study in China. Ecotoxicol. Environ. Saf..

[85] Qu Y., Lv Y., Ji S., Ding L., Zhao F., Zhu Y., Zhang W., Hu X., Lu Y., Li Y. (2022). Effect of exposures to mixtures of lead and various metals on hypertension, pre-hypertension, and blood pressure: A cross-sectional study from the China National Human Biomonitoring. Environ. Pollut..

[86] Fu Y., Liu Y., Liu Y., Wang Y., Zhu M., Lin W., Li M., Liu Y., He M., Yu L. (2022). Relationship between cumulative exposure to metal mixtures and heart rate among Chinese preschoolers. Chemosphere.

