# Supplementary material for: Urinary Levels of 14 Metal Elements in General Population: A Region-Based Exploratory Study in China
Source: Toxics. 2023 May 27;11(6):488. doi: 10.3390/toxics11060488 (PMC10302351; doi:10.3390/toxics11060488)
Supplement: Supplementary file 1 [file toxics-11-00488-s001.zip › toxics-2367553-supplementary.pdf]

Supporting Information

Urinary Levels of 14 Metal Elements in General Population: A Region-based Exploratory Study in China

Zining Zhang [a], Sai Guo [a], Liting Hua [a], Beibei Wang [b], Qiusheng Chen [c], Lu Liu [c], Li Xiang [d], Hongwen Sun [a] and Hongzhi Zhao [a,*]

[a] *Ministry of Education Key Laboratory of Pollution Processes and Environmental Criteria, College of Environmental Science and Engineering, Nankai University, Tianjin 300350, China*

[b] *School of Energy and Environmental Engineering, University of Science and Technology Beijing, Beijing 100083, China*

[c] *Institute of Agro-product Safety and Nutrition, Tianjin Academy of Agricultural Sciences, Tianjin, 300381, China*

[d] *State Key Laboratory of Environmental and Biological Analysis, Department of Chemistry, Hong Kong Baptist University, Hong Kong, China*

* Corresponding author: Corresponding author: Hongzhi Zhao

Tel: +86-22-23507800

Email: hongzhizhao@nankai.edu.cn

Contents

Table S1. Geometric means of urinary metal concentrations in different gender and age groups of participants.

Table S2. Levels of urinary elemental concentrations published by US Centers for Disease Control and Prevention.

Table S3. The distributions of urinary essential elements concentrations in other studies.

Table S4. The distributions of urinary non-essential elements concentrations in other studies.

Table S5. Correlation (Spearman) matrix for levels of 14 tested metals in Jingyuan County subjects.

Table S6. Different age groups urinary median concentrations (μg/L) of metals including Ni, As, Se, Tl, Sr, Pb, Cu and Zn.

Figure S1. The difference in urinary concentrations(μg/L) of essential metals (Cu: A, Fe: B, Se: C and Ni: D) between male and female.

Figure S2. The difference in urinary concentrations(μg/L) of non-essential metals (As: A, Sr: B, Sb: C, Cd: D, Pb: E, Cr: F, and Al: G) between male and female.

Figure S3. The difference in concentration ratio of Cu and Zn in urine between male and female.

Figure S4. The difference in concentration of Cr (A), Cd (B), Sb (C), Fe (D), Al (E) and Rb (F) in urine between different age groups.

Figure S5. The difference in concentration ratio of Cu and Zn in urine between different age groups.

Table S1. Geometric means of urinary metal concentrations in different gender and age groups of participants.

Urinary elements	LOD (μg/L)	LOQ (μg/L)	GM (μg/L) (95%CI)						
			All	Male	Female	Young children	Children	Adolescents	Adults
Cr	0.63	2.09	1.13 (0.99,1.30)	1.19 (0.92, 1.54)	1.08 (0.94, 1.24)	1.27 (1.04, 1.55)	1.74 (0.94, 3.21)	1.00 (0.78, 1.29)	0.99 (0.86, 1.16)
Ni	0.25	0.85	1.96 (1.59, 2.40)	2.15 (1.56, 2.96)	1.80 (1.38, 2.34)	3.03 (1.83, 5.03)	3.38 (2.10, 5.44)	2.93 (1.89, 4.55)	1.23 (0.95, 1.62)
As	0.01	0.04	24.58 (21.82, 27.70)	25.26 (21.36, 29.87)	24.01 (20.27, 28.44)	29.56 (21.29, 41.03)	31.48 (24.92, 39.76)	26.68 (20.43, 34.86)	20.81 (17.60, 24.68)
Se	0.76	2.55	16.4 (14.11, 18.95)	17.30 (13.80, 21.68)	15.58 (12.84, 18.91)	27.19 (17.60, 42.01)	24.70 (17.78, 34.31)	17.01 (12.44, 23.27)	12.03 (10.10, 14.47)
Sr	0.02	0.07	267.6 (234.7, 305.1)	247.5 (202.6, 302.3)	286.3 (241.0, 340.2)	294.7 (231.4, 375.3)	370.7 (293.6, 467.9)	244.0 (168.3, 353.7)	242.8 (199.7, 293.5)
Cd	0.01	0.04	0.53 (0.46, 0.62)	0.53 (0.42, 0.66)	0.54 (0.44, 0.65)	0.50 (0.34, 0.72)	0.57 (0.40, 0.80)	0.51 (0.37, 0.70)	0.54 (0.44, 0.68)
Sb	0.14	0.47	-	-	-	-	-	-	-
Tl	0.04	0.14	0.11 (0.09, 0.12)	0.09 (0.08, 0.11)	0.12 (0.10, 0.15)	0.09 (0.06, 0.13)	0.08 (0.06, 0.11)	0.10 (0.07, 0.14)	0.13 (0.11, 0.16)
Pb	1.77	5.91	-	-	-	-	-	-	-
Al	2.39	8.00	34.69 (31.03, 38.77)	31.11 (25.84, 37.47)	38.11 (31.79, 45.69)	34.62 (25.52, 46.98)	33.85 (23.53, 48.69)	38.58 (28.19, 52.78)	33.69 (28.04, 40.40)
Fe	1.44	4.80	38.36 (30 43, 39.53)	36.82 (27.09, 50.03)	39.75 (32.51, 48.61)	35.24 (25.47, 48.76)	46.96 (24.91, 88.53)	41.40 (27.08, 63.29)	36.21 (28.58, 45.12)
Cu	0.16	0.53	9.02 (8.28, 9.83)	9.26 (8.09, 10.61)	8.82 (7.90, 9.84)	11.90 (9.94, 14.24)	9.81 (7.98, 12.05)	9.21 (7.49, 11.32)	7.99 (7.10, 9.03)
Zn	0.40	1.33	397.0 (353.4, 446.0)	459.2 (388.1, 543.2)	350.1 (299.6, 409.0)	599.40 (501.0, 717.1)	579.7 (493.1, 681.6)	458.0 (353.7, 593.1)	296.2 (248.9, 348.0)
Rb	0.05	0.17	850.0 (763.4, 946.4)	777.3 (654.8, 922.7)	918.4 (804.2, 1049)	990.8 (818.6, 1199)	830.2 (669.6, 1029)	756.9 (583.4, 982.1)	844.5(719.7, 1009)

Table S2. Levels of urinary elemental concentrations published by US Centers for Disease Control and Prevention.

Urinary metal	Demographic Categories	Sampling Time (years)	N	GM (μg/L)	95th Percentile (μg/L)
As	Total population	2009-2010	2860	9.28	85.6
Cd	Total population	2017-2018	2808	0.132	0.832
Cr	Total population	2017-2018	2791	-	0.930
Pb	Total population	2015-2016	3061	0.284	1.26
Ni	Total population	2017-2018	2791	1.11	4.23
Sr	Total population	2015-2016	3061	85.5	299
Tl	Total population	2017-2018	2808	0.164	0.473

Table S3. The distributions of urinary essential elements concentrations in other studies.

Location	Subjects	N	Sampling Time (years)	Unit	Ni	Se	Fe	Cu	Zn	Rb
Guangzhou, Guangdong, China [a]	General population	480	2018	GM (μg/L)	2.25	35.4	—	—	—	—
Yunlin,Taiwan, China [b]	General population	2417	2009-2012, 2014-2015	GM (μg/gcreatinine)	11.63	—	—	18.13	—	—
Gongcheng Guangxi,China [c]	General population	2766	—	GM (μg/gcreatinine)	—	29.71	—	19.74	578.1	—
Shenzhen Guangdong, China [d]	General population	215	2011-2012, 2013-2014, 2015-2016	Median (μg/L)	—	26.1	65.1	31.3	617	—
Wuhan, Hubei, China [e]	Pregnant woman	598	2014-2015	Median (μg/L)	3.82	17.75	—	14.71	332.9	1878
Wuhan, Hubei, China [f]	Pregnant woman	113	2011-2012	GM (μg/L)	0.77	2.57	10.7	4.64	53.3	—
China [j]	Adults	11037	2017-2018	Median (μg/L)	1.74	16.08	—	—	—	—
China [h]	Children	456	—	GM (μg/gcreatinine)	2.87	24.57	31.81	9.37	484.2	1690
China [i]	Children	1061	2019	Mean (μg/g)	2.303	9.015	8.486	7.339	255.3	—
Berlin,German [j]	General population	77	—	GM (μg/gcreatinine)	1.4	—	—	—	288	—
Gothenburg,Sweden [k]	General population	60	—	GM (μg/gcreatinine)	0.86	13.2	2.55	5.13	207	—
Ethiopian [l]	General population	386	2015-2016	GM (μg/L)	6.95	—	—	5.21	283	494

[a] [1]; [b] [2]; [c] [3]; [d] [4]; [e] [5]; [f] [6]; [j] [7]; [h] [8]; [i] [9]; [j] [10]; [k] [11]; [l] [12]

Table S4. The distributions of urinary non-essential elements concentrations in other studies.

Location	Subjects	N	Sampling Time (years)	Unit	Cd	Sr	Sb	Tl	Pb	Al	As	Cr
Guangzhou, Guangdong, China [a]	General population	480	2018	GM (μg/L)	1.05	—	—	—	0.96	—	41	—
Yunlin,Taiwan, China [b]	General population	2417	2009-2012, 2014-2015	GM (μg/gcreatinine)	1.01	283.8	—	0.23	1.44	—	98.86	5.74
Gongcheng Guangxi,China [c]	General population	2766	—	GM (μg/gcreatinine)	—	114.0	—	—	—	—	41.39	—
Shenzhen Guangdong, China [d]	General population	215	2011-2012, 2013-2014, 2015-2016	Median (μg/L)	1.03	—	—		2.71	—	36.6	3.67
Wuhan, Hubei, China [e]	Pregnant woman	598	2014-2015	Median (μg/L)	0.79	239.7	—	0.54	—	40.76	24.98	1.38
Wuhan, Hubei, China [f]	Pregnant woman	113	2011-2012	GM (μg/L)	0.97	—	—	0.07	0.44	—	4.91	0.4
China [j]	Adults	11037	2017-2018	Median (μg/L)	0.64	—	0.05	0.27	1.05	—	20.8	57
China [h]	Children	456	—	GM (μg/gcreatinine)	0.087	68.62	0.136	0.095	0.462	13.89	21.45	0.307
China [i]	Children	1061	2019	Mean (μg/g)	0.32	108.1	—	—	1.5	26.08	—	0.61
Berlin,German [j]	General population	77	—	GM (μg/gcreatinine)	0.15	—	—	0.19	0.56	—	8.7	—
Gothenburg,Sweden [k]	General population	60	—	GM (μg/gcreatinine)	0.071	—	0.047	—	0.29	—	28.3	0.066
Ethiopian [l]	General population	386	2015-2016	GM (μg/L)	0.61	79.4	—	1.44	—	25	20.9	—

[a] [1]; [b] [2]; [c] [3]; [d] [4]; [e] [5]; [f] [6]; [j] [7]; [h] [8]; [i] [9]; [j] [10]; [k] [11]; [l] [12]

Table S5. Correlation (Spearman) matrix for levels of 14 tested metals in Jingyuan County subjects.

	Cr	Ni	As	Se	Sr	Cd	Sb	Tl	Pb	Al	Fe	Cu	Zn	Rb
Cr	1													
Ni	0.511**	1												
As	0.330**	0.484**	1											
Se	0.303**	0.441**	0.389**	1										
Sr	0.318**	0.282**	0.312**	0.194**	1									
Cd	0.316**	0.323**	0.328**	0.288**	0.297**	1								
Sb	0.515**	0.257**	0.195**	0.196**	0.273**	0.250**	1							
Tl	-0.087	-0.081	0.015	-0.024	-0.147*	-0.022	0.132	1						
Pb	0.634**	0.493**	0.363**	0.391**	0.270**	0.280**	0.491**	0.0160	1					
Al	0.272**	0.174*	0.184*	0.116	0.149*	0.116	0.491**	0.218**	0.340**	1				
Fe	0.447**	0.260**	0.152**	0.106	0.160*	0.142	0.467**	0.255**	0.426**	0.678**	1			
Cu	0.441**	0.438**	0.460**	0.428**	0.252**	0.408**	0.412**	0.177*	0.512**	0.405**	0.382**	1		
Zn	0.295**	0.372**	0.463**	0.497**	0.371**	0.357**	0.283**	-0.040	0.401**	0.257**	0.253**	0.568**	1	
Rb	0.308**	0.220**	0.325**	0.337**	0.003	0.371**	0.168*	0.339**	0.266**	0.263**	0.252**	0.464**	0.301**	1

*. Correlation is significant at the 0.05 level (2-tailed); **. Correlation is significant at the 0.01 level (2-tailed).

Table S6. Different age groups urinary median concentrations (μg/L) of metals including Ni, As, Se, Tl, Sr, Pb, Cu and Zn

Group	Ni (μg/L)	As (μg/L)	Se (μg/L)	Tl (μg/L)	Sr (μg/L)	Pb (μg/L)	Cu (μg/L)	Zn (μg/L)
Young children (1-8 years)	4.34	32.43	27.65	0.10	329.0	2.40	10.17	541.9
Children (6-11 years)	3.77	32.62	30.96	0.08	361.6	2.03	9.21	616.3
Adolescents (12-18 years)	3.36	26.27	20.00	0.12	270.3	2.27	10.19	507.1
Adults (>18 years)	1.56	22.71	13.87	0.13	266.1	1.56	7.38	285.6
p	<0.001	0.007	<0.001	0.023	0.046	0.014	0.001	<0.001

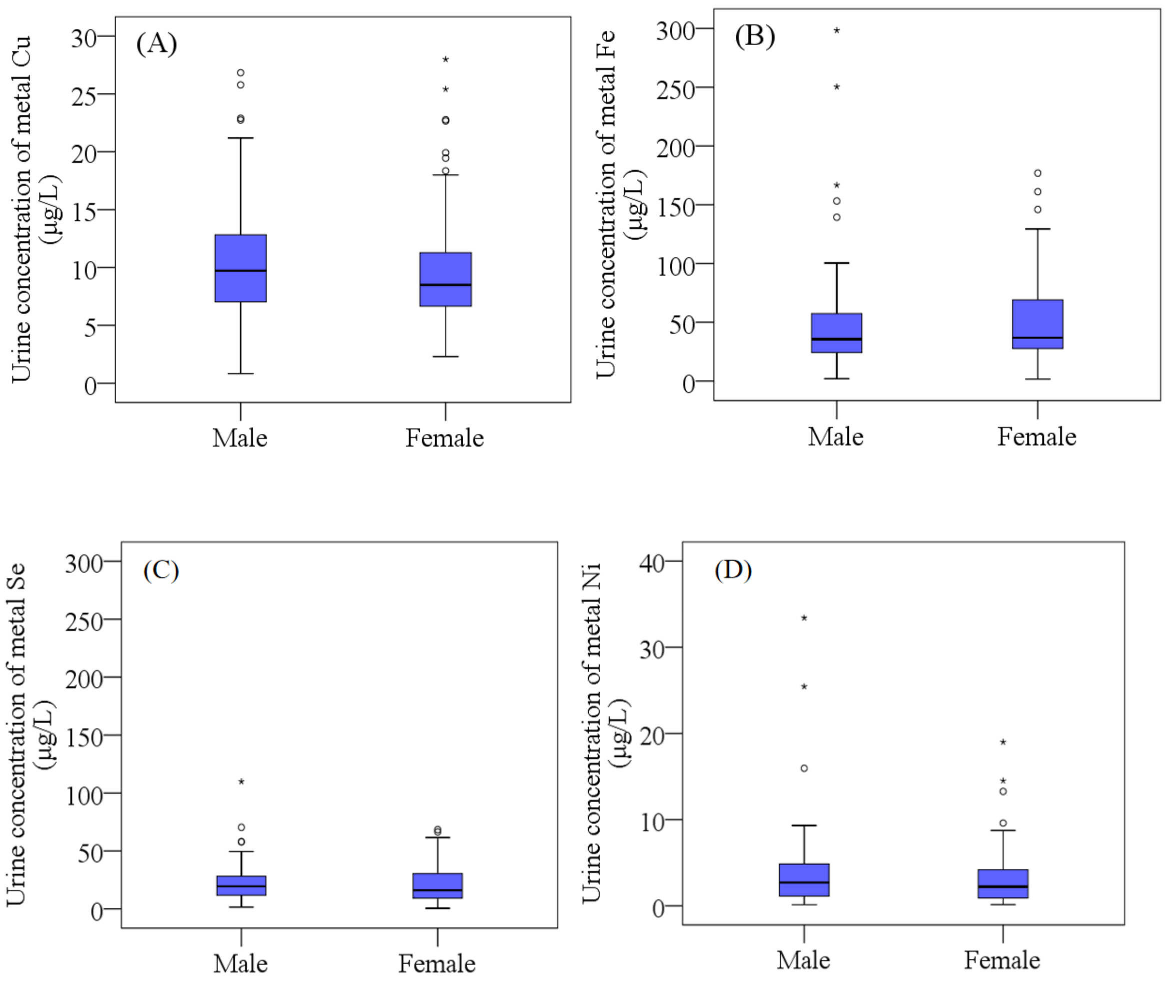

Figure S1. The difference in urinary concentrations (μg/L) of essential metals (Cu: A, Fe: B, Se: C and Ni: D) between male and female (There were no statistically significant difference between the above elements in each group; $p>0.05$). I: Range within 1.5 IQR. —: Median line. ○: Outliers. *: Extreme cases.

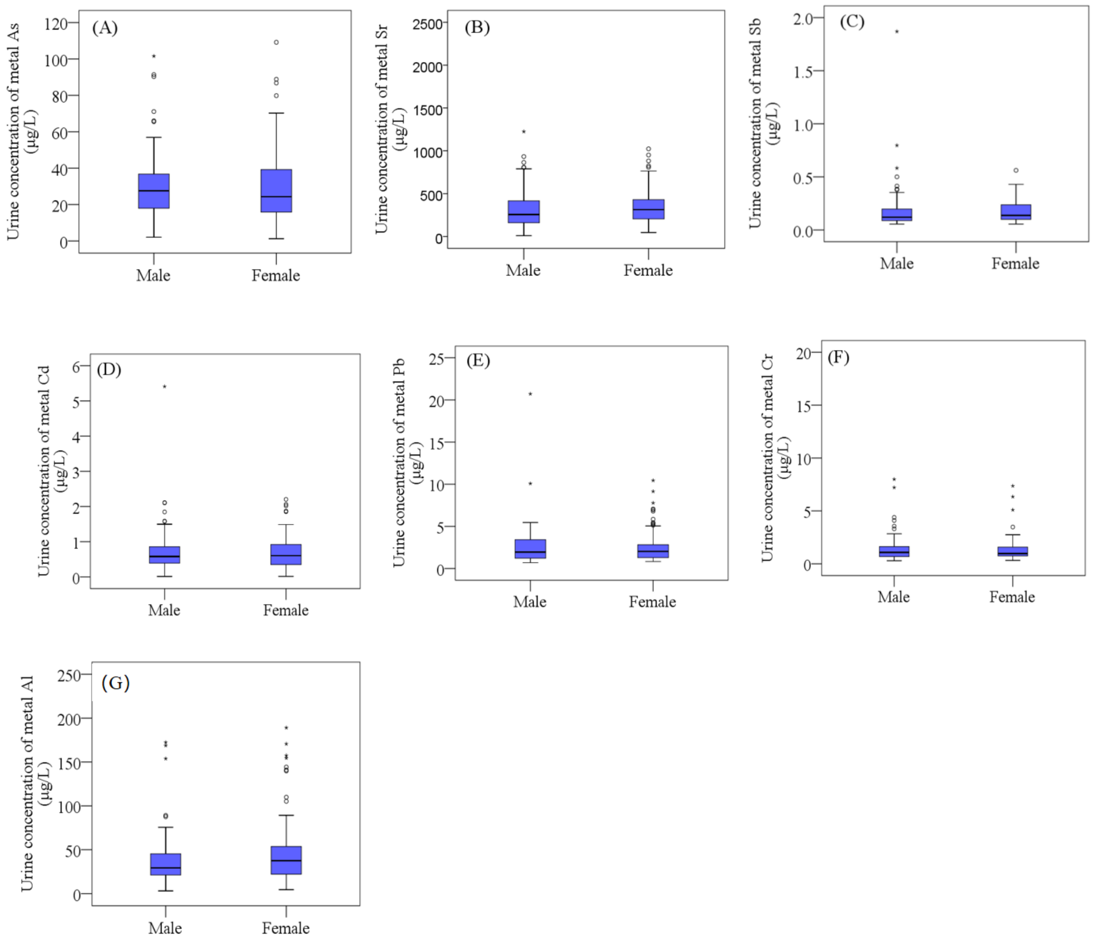

Figure S2. The difference in urinary concentrations (μg/L) of non-essential metals (As: A, Sr: B, Sb: C, Cd: D, Pb: E, Cr: F, and Al: G) between male and female (There were no statistically significant difference between the above elements in each group; *p*>0.05). I: Range within 1.5 IQR. ─: Median line. ○: Outliers. *: Extreme cases.

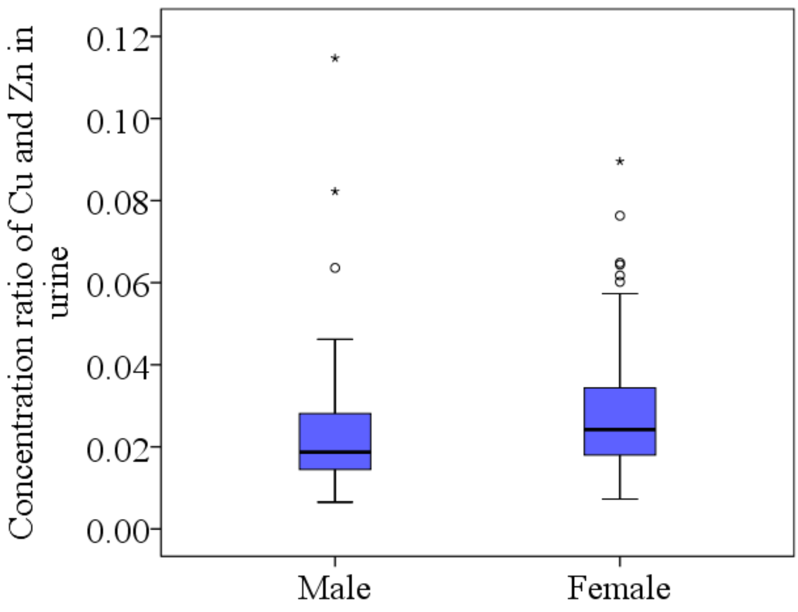

Figure S3. The difference in concentration ratio of Cu and Zn in urine between male and female (p<0.05). Ɪ: Range within 1.5 IQR. ─: Median line. ○: Outliers. *: Extreme cases.

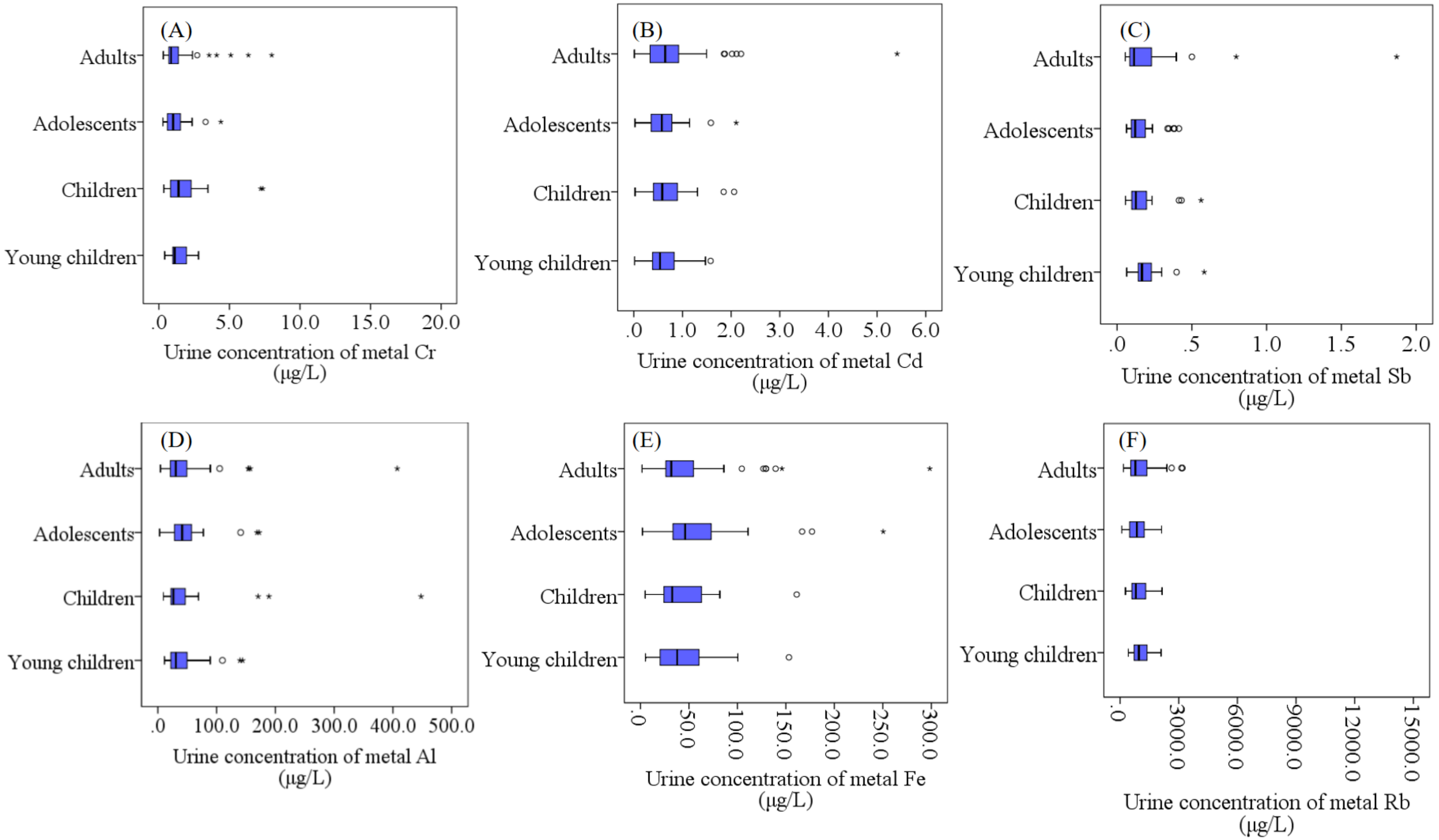

Figure S4. The difference in concentration of Cr (A), Cd (B), Sb (C), Fe (D), Al (E) and Rb (F) in urine between different age groups (There were no statistically significant difference between the above elements in each group; *p*>0.05).

Ɪ: Range within 1.5 IQR. ─: Median line. ○: Outliers. *: Extreme cases.

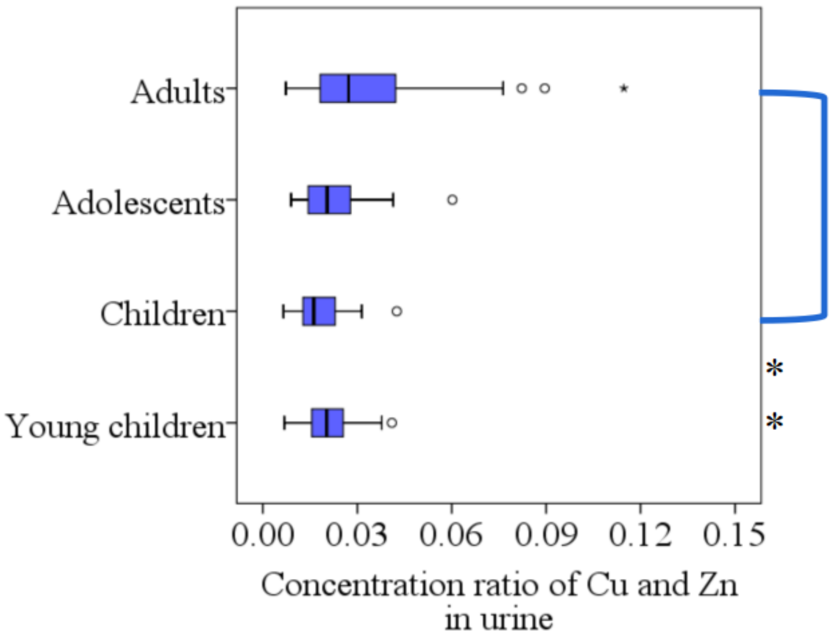

Figure S5. The difference in concentration ratio of Cu and Zn in urine between different age groups (p<0.001). Inside boxplots: I: Range within 1.5. Interquartile range (IQR).─: Medianline. ○: Outlier. *: Extreme cases. Outside boxplots:]: Significant differences between the two groups. ∗∗: p<0.01.

References

1. Zhong, Z.; Li, Q.; Guo, C.; Zhong, Y.; Zhou, J.; Li, X.; Wang, D.; Yu, Y. Urinary heavy metals in residents from a typical city in South China: human exposure and health risks. *Environmental Science and Pollution Research* **2022**, *29*, 15827-15837, doi:10.1007/s11356-021-16954-0.
2. Lee, N.; Wang, H.; Du, C.; Yuan, T.-H.; Chen, C.; Yu, C.; Chan, C. Air-polluted environmental heavy metal exposure increase lung cancer incidence and mortality: A population-based longitudinal cohort study. *Science of the Total Environment* **2022**, *810*, 152186, doi:10.1016/j.scitotenv.2021.152186.
3. Mo, X.; Cai, J.; Lin, Y.; Liu, Q.; Xu, M.; Zhang, J.; Liu, S.; Wei, C.; Wei, Y.; Huang, S.; et al. Correlation between urinary contents of some metals and fasting plasma glucose levels: A cross-sectional study in China. *Ecotoxicology and Environmental Safety* **2021**, *228*, 152186, doi:10.1016/j.ecoenv.2021.112976.
4. Yang, D.; Liu, Y.; Liu, S.; Li, C.; Zhao, Y.; Li, L.; Lu, S. Exposure to heavy metals and its association with DNA oxidative damage in municipal waste incinerator workers in Shenzhen, China. *Chemosphere* **2020**, *250*, 126289, doi:10.1016/j.chemosphere.2020.126289.
5. Fang, X.; Qu, J.; Huan, S.; Sun, X.; Li, J.; Liu, Q.; Jin, S.; Xia, W.; Xu, S.; Wu, Y.; et al. Associations of urine metals and metal mixtures during pregnancy with cord serum vitamin D Levels: A prospective cohort study with repeated measurements of maternal urinary metal concentrations. *Environment International* **2021**, *155*, 106660, doi:https://doi.org/10.1016/j.envint.2021.106660.
6. Zhang, M.; Liu, C.; Li, W.; Xu, X.; Cui, F.; Chen, P.; Deng, Y.; Miao, Y.; Luo, Q.; Zeng, J.; et al. Individual and mixtures of metal exposures in associations with biomarkers of oxidative stress and global DNA methylation among pregnant women. *Chemosphere* **2022**, *293*, 133662, doi:10.1016/j.chemosphere.2022.133662.
7. Qu, Y.; Lv, Y.; Ji, S.; Ding, L.; Zhao, F.; Zhu, Y.; Zhang, W.; Hu, X.; Lu, Y.; Li, Y.; et al. Effect of exposures to mixtures of lead and various metals on hypertension, pre-hypertension, and blood pressure: A cross-sectional study from the China National Human Biomonitoring. *Environmental Pollution* **2022**, *299*, 118864, doi:10.1016/j.envpol.2022.118864.
8. Xue, Q.; Zhou, Y.; Gu, H.; Xie, X.; Hou, F.; Liu, Q.; Wu, H.; Zhu, K.; Wan, Z.; Song, R. Urine metals concentrations and dyslexia among children in China. *Environment International* **2020**, *139*, 105707, doi:10.1016/j.envint.2020.105707.
9. Fu, Y.; Liu, Y.; Liu, Y.; Wang, Y.; Zhu, M.; Lin, W.; Li, M.; Liu, Y.; He, M.; Yu, L.; et al. Relationship between cumulative exposure to metal mixtures and heart rate among Chinese preschoolers. *Chemosphere* **2022**, *300*, 134548, doi:10.1016/j.chemosphere.2022.134548.
10. Schmied, A.; Murawski, A.; Kolossa-Gehring, M.; Kujath, P. Determination of trace elements in urine by inductively coupled plasma-tandem mass spectrometry - Biomonitoring of adults in the German capital region. *Chemosphere* **2021**, *285*, 131425, doi:10.1016/j.chemosphere.2021.131425.
11. Barregard, L.; Ellingsen, D.G.; Berlinger, B.; Weinbruch, S.; Harari, F.; Sallsten, G. Normal variability of 22 elements in 24-hour urine samples - Results from a biobank from healthy non-smoking adults. *International Journal of Hygiene and Environmental Health* **2021**, *233*, 113693, doi:10.1016/j.ijheh.2021.113693.
12. Godebo, T.; Paul, C.J.; Jeuland, M.A.; Tekle-Haimanot, R. Biomonitoring of metals and trace elements in urine of central Ethiopian populations. *International Journal of Hygiene and Environmental Health* **2019**, *222*, 410-418, doi:10.1016/j.ijheh.2018.12.007.
